# Statue of Bichat, at Bourg

**Published:** 1843-10-28

**Authors:** 


THE MEDICAL EXAMINER.

PHILADELPHIA, OCT. 28, 1843.

### INAUGURATION OF THE STATUE OF BICHAT, AT BOURG.

A magnificent statue, erected to the memory of the illustrious Bichat, at Bourg, the capital of the Department of Ain, in the south-east of France, was inaugurated, with great pomp and ceremony, on the 24th of August last. The statue, which is by M. David, of Angers, is in bronze, and represents Bichat fixing his gaze on a living infant, while at his feet is a dead body, partially dissected, ingeniously allegorical of his celebrated work on "*Life and Death.*" The departmental authorities, and those of the town, assisted at the ceremony, and the statue was unveiled amidst a salvo of artillery. Among the distinguished medical notabilities present, were M. Pariset, the Perpetual Secretary of the French Academy of Medicine, M. Royer-Collard, on the part of the Medical Faculty of Paris, M. Hypolite Larrey, as representative of the *Societè Medicale d'Emulation de Paris,* of which Bichat was the founder; with others from Lyons, &c. The brother and nephew of Bichat, with several other relatives, were also present. The above gentlemen all pronounced *Eloges;* individually and collectively they are bad; rhetorical flourishes, unmeaning rhapsodies, or dull, stupid details.

Whilst this magnificent ceremony was passing in an obscure town in France, his tomb at Paris is in a lamentable condition. Dr. Londe, at a recent meeting of the Academy of Medicine, stated that having had his attention called to the subject, he had visited the spot where Bichat was buried, accompanied by two friends, who were present at the interment. A stone placed against the wall, in the old Cemetery of Saint Catharine, at Clamart, with the name of Bichat half effaced, alone indicates the spot. This cemetery has been disposed of by the city of Paris, so that, unless some measures are speedily taken, the remains of the illustrious founder of General Anatomy will repose in a ditch. A proposition was made to the Academy to purchase sufficient ground at Pére la Chaise, deposit there the remains of the great philosopher, and erect over them a plain monument. Several of the members were anxious that the remains should be transported to Bourg, and deposited beneath the monument; but it was very properly answered, that the reputation of Bichat was made at Paris, and that at Paris he should be buried.

